# Application of transposon systems in the transgenesis of bovine somatic and germ cells

**DOI:** 10.1186/s12917-022-03252-1

**Published:** 2022-04-27

**Authors:** Dong-Hyeok Kwon, Gyeong-Min Gim, Kyeong-Hyeon Eom, Ji-Hyun Lee, Goo Jang

**Affiliations:** 1 Laboratory of Theriogenology, College of Veterinary Medicine, Seoul National University, Gwanak-ro, Gwanak-gu, Seoul, Republic of Korea 08826; 2 BK21 PLUS program, College of Veterinary Medicine, Seoul National University, Seoul, 08826 Republic of Korea; 3 LARTBio Inc, Seoul, Republic of Korea

**Keywords:** Cattle, Electroporation, Microinjection, Transposons, Transgenesis

## Abstract

**Background:**

Several DNA transposons including *PiggyBac* (PB), *Sleeping Beauty* (SB), and Tol2 have been applied as effective means for of transgenesis in many species. Cattle are not typically experimental animals, and relatively little verification has been presented on this species. Thus, the goal here was to determine the applicability of three transposon systems in somatic and embryo cells in cattle, while also investigating which of the three systems is appropriate for each cell type. Green fluorescent protein (GFP)-expressing transposon systems were used for electroporation and microinjection in the somatic cells and embryo stage, respectively. After transfection, the GFP-positive cells or blastocysts were observed through fluorescence, while the transfection efficiency was calculated by FACS.

**Results:**

In bovine somatic cells, the PB (63.97 ± 11.56) showed the highest efficiency of the three systems (SB: 50.74 ± 13.02 and Tol2: 16.55 ± 5.96). Conversely, Tol2 (75.00%) and SB (70.00%) presented a higher tendency in the embryonic cells compared to PB (42.86%).

**Conclusions:**

These results demonstrate that these three transposon systems can be used in bovine somatic cells and embryos as gene engineering experimental methods. Moreover, they demonstrate which type of transposon system to apply depending on the cell type.

**Supplementary Information:**

The online version contains supplementary material available at 10.1186/s12917-022-03252-1.

## Highlights

- Non-viral methods for genome integration of the gene-of-interest.
- Potential Tol2 transposon system for bovine somatic cell and embryo transfection.
- PiggyBac transposon system for electroporation-mediated transfection in somatic cells.
- Sleeping Beauty and Tol2 systems for microinjection-mediated transfection in embryos.
- Applicability of three transposon systems in the transgenesis of bovine cells and embryos.

## Background

Genomic engineering approaches, such as transgenesis are largely divided into viral and non-viral vector methods. Although viral methods provide the advantage of high transfection efficiency, their disadvantages include immunogenic and oncogenic side effects alongside providing limited vector capacities. Alternatively, non-viral methods offer a relatively low immune response and permit the introduction of a moderately larger exogenous DNA. However, they exhibit lower transfection efficiencies compared to viral methods [1, 2]. The DNA transposons used in these non-viral vector methods are mobile jumping genes, which form large portions of the mouse, human, rat, *E. coli*, plants like maize, and zebrafish genomes [3–5]. Transposons have been continuously used as non-viral gene-editing methods for integration into the genome, which occurs through the recognition of specific sequences by the corresponding transposases, according to the type of transposon. During the DNA transposon integration, transposases bind to Terminal Inverted Repeats (TIRs) and cut-and-paste to specific target sequences [6]. The transposon method also allows stable expression of the introduced exogenous genes by causing random site preferred integration in genomes and with stability in favor of low-risk chromosomal intron integration [6, 7].

Among the transposon systems, *PiggyBac* (PB), *Sleeping Beauty* (SB), and Tol2 have been used as three main transposons within vertebrates [8–13]. These three transposon methods have been validated in several species, such as rodents, zebrafish, and human cells, and have been mainly used in studies related to overexpression and therapeutic approaches [8, 14–16]. Moreover, cows possess 80% genome consistency, can be used as a potential alternative model for human disease research, and demonstrate low alternative splicing (AS), which is a critical process for changing the genomic instruction into functional proteins. However, in contrast to rodents, the application of transposon systems has not been sufficiently investigated in large animals [17]. Accordingly, this study investigates which transposon could effectively deliver and integrate transgenes to bovine somatic cells or embryos. Therefore, these methods could potentially be applied to produce genetically modified cattle models in the future.

## Results

### Delivery of transposon systems to bovine somatic cells by electroporation

To determine the initial and integration transfection efficiencies of the three transposons, GFP expression ratio was analyzed by FACS on day 3 and 10 post-transfection, respectively. FACS analysis on day 3 (Supplementary Fig. 1) post-transfection showed significant differences in PB compared to the SB and Tol2 systems, whereby PB presented the highest transfection efficiency (Fig. 2B, PB: 98.37 ± 1.29, SB: 63.43 ± 13.84, and Tol2: 61.57 ± 5.68, *p* < 0.05). Similarly, PB exhibited a higher integration efficiency than SB and Tol2 in the day 10 (Supplementary Fig. 2) post-transfection re-sorting results (Fig. 2C, PB: 63.97 ± 11.56, SB: 50.74 ± 13.02 and Tol2: 16.55 ± 5.96, *p* < 0.05).

### Application of transposon systems in bovine embryo cytoplasmic microinjection

No significant differences were observed in the developmental competence at the 8-cells and blastocysts formation following the microinjection of two different DNA concentrations (50 ng/μL vs 25 ng/μL; Table 1). Similarly, no differences were found in the total cell numbers between all the blastocysts derived from the microinjections (Table. 1). However, the ratio of GFP expression in blastocysts of the SB microinjection group at 50 ng/μL was higher (66.70%) than in the other two transposons (Tol2: 47.80%, PB: 35.30%). Although, no significant difference was noted. Finally, SB (70.00%) and Tol2 (75.00%) had a higher expression tendency than PB (42.86%) in the 25 ng/μL microinjection trial (Table 1; *P* > 0.05).

**Table 1:** Cytoplasmic microinjection efficiency of transposon systems in bovine embryos

Concentration	IVM	Microinjection	IVC			
High^a^	No. COCs	Condition	No. 8-cells (%)	Total Blastocysts (%)	GFP Expressing Blastocysts (%)	Total Cell Number
	122	Wild type	79 (64.8)	41 (33.6)	0 (0)	92.64 ± 23.69
	130	PiggyBac	60 (46.2)	17 (13.1)	6 (35.3)	89.20 ± 14.82
	130	Sleeping Beauty	51 (39.2)	15 (11.5)	10 (66.7)	86.40 ± 23.52
	130	Tol2	67 (51.5)	23 (17.7)	11 (47.8)	96.00 ± 18.58
Low^b^	No. COCs	Condition	No. 8-cells (%)	Total Blastocysts (%)	GFP Expressing Blastocysts (%)	Total Cell Number
	78	Wild type	51 (65.38)	21 (26.92)	0 (0)	100.00 ± 33.08
	77	PiggyBac	31 (40.26)	14 (18.18)	6 (42.86)	95.50 ± 25.03
	77	Sleeping Beauty	30 (38.96)	10 (12.99)	7 (70.00)	102.00 ± 28.58
	77	Tol2	31 (40.26)	15 (15.58)	9 (75.00)	100.78 ± 24.99

DNA concentrations were described as High and Low (High: 50 ng/μL, Low: 25 ng/μL). Wild type condition means untreated standard in vitro production embryo

The percentage of GFP expressing blastocyst was calculated as the number of GFP expressing blastocysts out of the total number of blastocysts

*IVM* In vitro maturation, *IVC* In vitro culture, *COC* Cumulus-oocyte complex

^a^High: Mixture containing 50 ng/μL of the transposon plasmid along with 50 ng/ μL of the transposase plasmid

^b^Low: A mixture containing 25 ng/μL of the transposon plasmid and 25 ng/μL of the transposase plasmid

## Discussion

Here, electroporation was used on three transposon systems to determine whether any could be used as a stable gene engineering tool in bovine somatic cells. Following verification in somatic cells, embryo microinjection was performed to similarly verify that these systems worked reliably in germline cells. Firstly, in the bovine somatic cells, it was difficult to guarantee high efficiency and survival using primary cultured somatic cells instead of immortalized cell lines [18]. Therefore, methods that introduce viruses such as the adeno-associated virus (AAV), retrovirus, and lentivirus were implemented. However, this viral method presents a major disadvantage, whereby it promotes numerous side effects such as triggering an immune response and tumor formation, while it also has difficulty in accompanying large plasmids [19]. Our study shows that each transposon system could be applied in the introduction of genes using electroporation methods as an alternative to the viral methods. Additionally, a PB-based transfection revealed higher gene transfection and integration efficiencies, despite the delivery of genes to primary cells.

In the embryo, cytoplasmic microinjection was utilized to deliver exogenous transposon system plasmids. This method of microinjection is easy to perform, avoids direct damage to the nucleus, and provides higher embryonic viability than pronuclear microinjections. Moreover, most livestock animal zygotes such as pigs, sheep, and cattle are comprised of a high composition of fatty acids, which makes the cytoplasm dark and thus difficult to find the pronuclear [20–22]. Here, the injection concentration was divided into both high and low concentrations to assess the transposon systems at the germ cell stage. The injection efficiency varied greatly depending on the concentration. In follow-up experiments that use the bovine cytoplasmic injection, the concentration optimization will be performed according to each transposon system. Contrary to the results in the somatic cell, SB and Tol2 showed a higher frequency of GFP expression than the PB system. These differences are likely due to the somatic and germline cells being distinctly different cells, as well as the different methods of transfection that were used for each cell, namely, the electroporation of the somatic cell and microinjection of the germ cell [23]. Additionally, there were no significant differences between the embryo microinjection tests a variation in the quality of ovaries received for each experiment, alongside different IVF and S-phase timing [23].

The vectors applied in the transposon experiments did not exceed 10 kb. In further studies, it will be necessary to investigate whether transposon vectors larger than 10 kb affect the efficiency of each transposon system [24]. Moreover, the transposon represents a further method of introducing exogenous coding sequences that randomly integrate into genomes. Therefore, future research requires the study of the number of copies inserted into external genes, introduced genetic loci, stability, and changes in genes associated with cellular and embryonic development, in addition to, gene introduction and integration efficiency.

Furthermore, each transposon recognizes a specific target sequence during random integration into the genome. For PB, SB, and Tol2, the integration occurs by recognizing TTAA, TA, and heterogenic sequences of 8 bp in length, respectively [6]. Therefore, various characteristics are divided according to the type of transposon. Previous in vivo experiments in our laboratory have produced transgenic cattle using PB and SB, which have shown germline transmission for more than 6 years without health problems [25]. The embryo results of this experiment demonstrate that PB, SB, and Tol2 are highly efficient and that Tol2 can be used as an alternative to PB and SB.

Double or sandwich transposon methods were discovered and provide more efficient and powerful transposon systems. In further studies, the development of three fusion transposon systems could be a key factor in efficient DNA delivery; particularly with a PB-SB-Tol2 fused form of the transposon, which could compensate for the shortcomings of each transposon alone [26, 27]. In conclusion, these data demonstrated that all three transposons (PB, SB, and Tol2) promoted stable expression of exogenous genes without silence in long-term culture. On a practical level, we suggest that PB is preferred for gene delivery and SB for embryonic levels for bovine genomic studies.

## Conclusion

As cows present an 80% genome competency with humans and low alternative splicing (AS), they represent a suitable model for human disease and transgenesis. This paper used three transposon systems (PB, SB, and Tol2) in other species that were applied for the non-viral genome integration method in bovine somatic and germ cells. Overall, our findings indicate that all systems have the possibility of application in both bovine somatic and germ cells, in addition to, highlighting which transposon represents the appropriate method for each cell type.

## Methods

### DNA construction

**Fig. 1:**
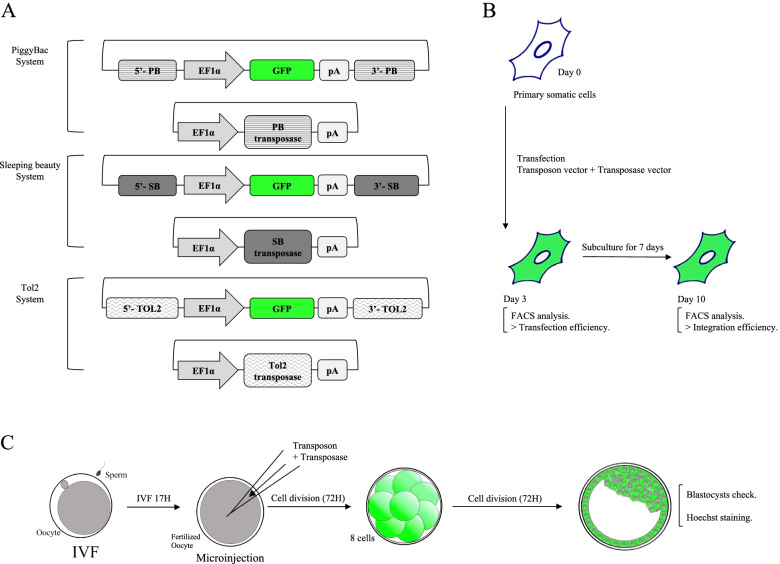
Schematic design of the transposon systems (PB, SB, and Tol2) application in bovine somatic and germ cells. **A** Illustration of the transposon DNA composition including the Ef1α promoter and GFP reporter gene. **B** Transfection and analysis method for the somatic cells, and **C**) germ cells

In previous studies, the transposon system vectors for SB (pCMV (CAT)T7-SB100X) and PB (pCy43 and PB-CA) were purchased from Addgene (http://www.addgene.org, Plasmids #34879 and #20960, respectively), and/or provided by the Sanger Institute (Hinxton, UK) for the PB and SB systems [28, 29]. To establish a novel Tol2 system, the transposon and transposase were purchased from Addgene (http://www.addgene.org, Plasmids #97151 and #31823, respectively). To avoid the potential backbone sequences effect in the application of the transposon systems, all transposon element sequences were amplified by PCR, and the PCR products were run for 15 min before being extracted with a Qiagen Gel Extraction Kit (Cat No. 28704). The extracted PCR products were cloned with a Qiagen TA cloning kit (Cat No. 231124) (Fig. 1A). All the reconstructed vectors were sequenced fully.

### Primary cell culture

The bovine somatic cells were isolated from three types of ear skin, which were taken from newborn calves using a biopsy punch. After isolation, tissues were collected directly into a 50 mL conical tube containing 5 mL of 10% penicillin in PBS to prevent contamination and moved to a bench in 3 h. The tissues were washed 3 times with 10% penicillin-PBS and minced on 100 mm Petri dishes (Falcon, Cat No. 351029) for 5 min, before collection in 15 mL conical tubes (SPL, Cat No. 50015) with 5 mL of 10% penicillin-PBS. After centrifugation at 13,000 rpm for 3 min, the supernatant was aspirated off and then the pellet was washed with 5 mL of the 10% penicillin-PBS solution. After 3 cycles of the centrifugation to washing steps, the pellet was resuspended with 10 mL of HBSS containing 1% collagenase and incubated for 17 h at 37 °C with 5% CO_2_. Thereafter, the samples were centrifuged at 13,000 rpm for 3 min and the pellet was resuspended with DMEM (HyClone, Cat No. SH30243.01, USA) containing 20% FBS (Gibco, Cat No. 26140079, USA). The resuspended samples were seeded into a 60 mm dish for further cell culture.

### Cell transfection (electroporation) and FACS sorting

Three types of primary cells were used for the electroporation-mediated DNA transfections through the Neon® Transfection system (Invitrogen Cat No. MPK5000). A Cell Countess II Automated Cell Counter (Thermo Fisher Scientific) was used to count 3 × 10^5^ cells for transfection. The transfection was replicated 3 times per cell type and the transfection conditions were optimization no.16 (1400 V, 20 ms, and 2 pulses). Post-transfection, the cells were seeded directly into incubated 6-well plates containing 3 mL DMEM. The culture media was changed to fresh media seventeen hours later to remove any dead cells (Fig. 1B).

**Fig. 2:**
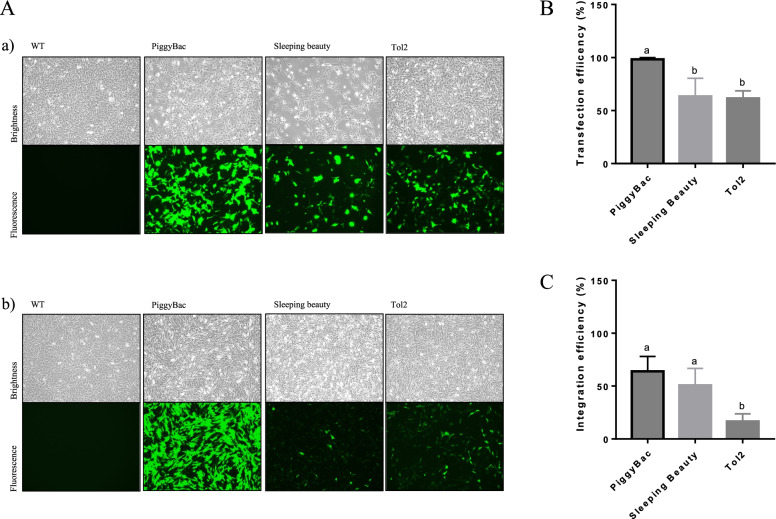
Transposon system-mediated bovine somatic cell transfection using electroporation and FACS analysis to examine the transfection and integration efficiencies. All transfections were replicated 3 times with 3 types of primary cells. **A**) A representative bright and GFP fluorescent field for each transposon system, A-a and **B**) a representative image and FACS result for the transfection efficiency on day 3; A-b and **C**) the integration efficiency on day 10 after transfection

FACS analysis was conducted to measure the transfection efficiency and integration. To measure the transfection efficiency, FACS was conducted 3 days following transfections (Fig. 2 A. [a]). The integration efficiency was measured on day 10 after the day-3 sorted cells were sub-cultured for an additional 7 days (Fig. 2 A. [b]). To conduct FACS sorting, transfected cells were suspended with 500 μL of PBS and the samples were analyzed on a BD Bioscience FACS Aria II, installed at the National Center for Interuniversity Research Facilities (NCIRF) at Seoul National University.

### In vitro maturation of bovine immature oocytes

Ovaries were collected from a local slaughterhouse within 2–3 h of removal and transported in 0.9% saline solution at 30–35 °C to the laboratory. Cumulus–oocyte complexes (COCs) from follicles 2 to 8 mm in diameter were aspirated using an 18-gauge needle, selected, and collected in a 10-cm Petri dish. The residue was washed 3 times with HEPES-buffered tissue culture medium-199 (TCM-199; Invitrogen, Carlsbad, CA, USA) supplemented with 2 mM NaHCO_3_ (Sigma–Aldrich Corp., St. Louis, MO, USA), 10% FBS and 1% penicillin–streptomycin (v/v). For in vitro maturation, COCs were cultured in four-well dishes (30–40 oocytes per well; Falcon, Becton-Dickinson Ltd., Plymouth, UK) for 22–24 h in 450 μL TCM-199 supplemented with 0.005 AU/mL FSH (Sigma–Aldrich), 10% FBS, 1 μg/mL 17β-estradiol (Sigma–Aldrich) and 100 μM Cysteamine (Sigma-Aldrich) at 38.5 °C under 5% CO_2_.

### Sperm preparation, in vitro fertilization (IVF), and in vitro culture of embryos (IVC)

The Percoll gradient method used for the separation and purification of motile spermatozoa has been previously described [30]. Briefly, spermatozoa were refined from thawed semen straws by density-gradient centrifugation on a Percoll discontinuous gradient (45–90%) at 1680 rpm for 15 min. For the Percoll density gradient, a 45% Percoll solution was prepared with 1 mL of 90% Percoll (Nutricell, Campinas, SP, Brazil) and 1 mL of capacitation-TALP (Nutricell) [31]. Thereafter 1 mL of 45% Percoll solution was added to 1 mL of 90% Percoll solution in a 15 mL conical tube. The thawed semen was layered onto the top of the Percoll gradient solution, and the tube was centrifuged. The pellet was washed twice with 3 mL of TALP by pipetting before being centrifuged at 1680 rpm for 5 min. The pelleted active and motile spermatozoa were added to droplets containing matured oocytes. Oocytes were inseminated on (day 0) with 1–2 × 10^6^ spermatozoa/mL for 17 h in IVF-TALP medium (Nutricell) under NidOil (Nidacon). The fertilized zygotes were denuded and cultured in a two-step defined culture medium (4 days in D1 medium before transfer to D2 medium) at 38.5 °C in an atmosphere of 5% O_2_, 5% CO_2_, and 90% N_2_ [32]. The cleavage rates were recorded on day 4, while the embryonic development was monitored according to the stages of the International Embryo Transfer Society (IETS).

### Microinjection

**Fig. 3:**
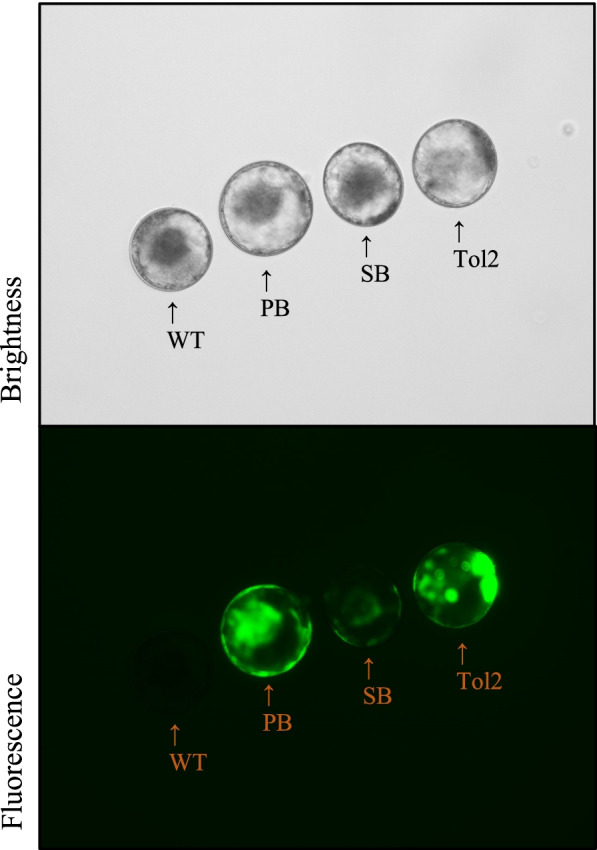
Representative bright and GFP fluorescent field of each transposon system in bovine blastocysts. Blastocysts were observed through a fluorescent microscope on day 7

Microinjection experiments were conducted through three different transposon systems to analyze whether the stable transposon systems in somatic cells could be used at the bovine embryo stage. The denuded zygotes were used for microinjection (Femtojet®, Eppendorf, Germany) following 17 h of IVF. To determine the optimal microinjection conditions, 2 different DNA concentrations were assessed (High: Mixture containing 50 ng/μL of the transposon plasmid along with 50 ng/ μL of the transposase plasmid. Low: A mixture containing 25 ng/μL of the transposon plasmid and 25 ng/μL of the transposase plasmid). The GFP-expressing blastocysts were observed with a fluorescent microscope after 6 days (Figs. 1 C, 3, and Table 1).

### Hoechst 33342 staining and total cell counting

The blastocysts on day 7 were washed twice with HEPES-buffered tissue culture medium-199 (TCM-199; Invitrogen, Carlsbad, CA, USA) supplemented with 2 mM NaHCO_3_ (Sigma–Aldrich Corp., St. Louis, MO, USA), 10% FBS and 1% penicillin–streptomycin (v/v) and stained for 4 min with Hoechst 33342. After staining, the blastocysts were washed twice with PBS and mounted on a glass slide. Hoechst 33342 stained cells were observed through a fluorescence microscope and manually counted using Image J software (NIH) (Supplementary Fig. 3).

### Statistical analysis

Data were obtained from three replicated experiments and statistically analyzed using a one-way ANOVA with a Tukey’s multiple comparisons test, which were performed using GraphPad Prism version 7.0.0 for Windows, GraphPad Software, San Diego, California USA, www.graphpad.com. The results were considered statistically significant when the *p* value was equal to or lower than 0.05.

## Supplementary Information

**Additional file 1.**

## Data Availability

The datasets used and/or analyzed during the current study are available from the corresponding author upon reasonable request.

## Abbreviations

PB: PiggyBac
SB: Sleeping Beauty
GFP: Green fluorescent protein
FACS: Fluorescence-activated cell sorting
TIRs: Terminal Inverted Repeats
AS: Alternative Splicing
AAV: Adeno-associated virus
PBS: Phosphate-buffered saline
DMEM: Dulbecco’s modified eagle’s medium
COCs: Cumulus–oocyte complexes
IVM: In vitro maturation
IVF: In vitro fertilization
IVC: In vitro culture
