# Supplementary material for: Application of transposon systems in the transgenesis of bovine somatic and germ cells
Source: BMC Vet Res. 2022 Apr 27;18:156. doi: 10.1186/s12917-022-03252-1 (PMC9044889; doi:10.1186/s12917-022-03252-1)

Supplementary Fig. 1. Representative FACS assay results on Day 3 after transfection in bovine somatic cells. A) Wild type, B) PB, C) SB and D) Tol2 transposon system. P4 area shows GFP positive cells except for doublet and dead cells. P4 area cells are totally sorted for subculture to examine integration rate.

Supplementary Fig. 2. Representative FACS assay results on Day 10 after integration in bovine somatic cells. A) Wild type, B) PB, C) SB and D) Tol2 transposon system. P4 area shows GFP positive cells except for doublet and dead cells.

Supplementary Fig. 3. Representative images of Hoechst staining and total cell counting in transposon-mediated GFP expressing bovine embryos. Total cell number was counted manually using Image J software (NIH). A-A’) Wild type, B-B’) PB, C-C’) SB and D-D') Tol2 transposon system.

Supplementary Fig. 1.

A)


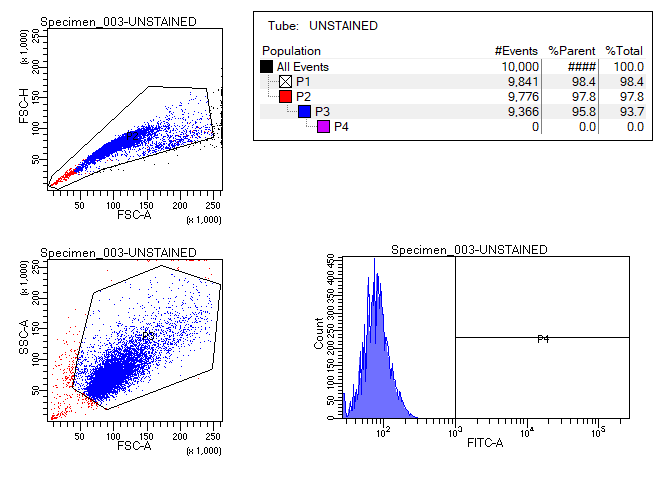


B)


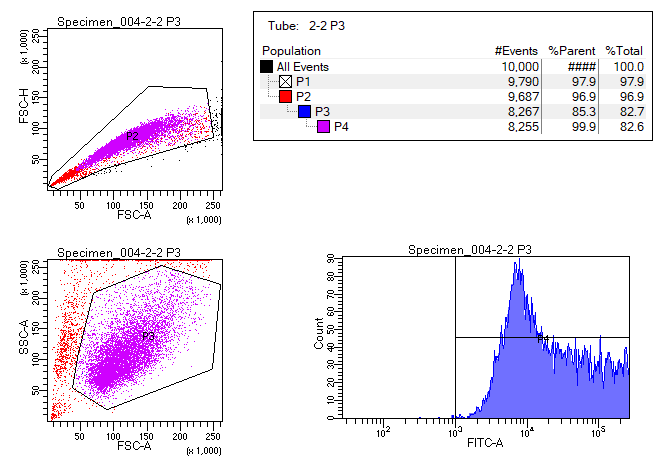


C)


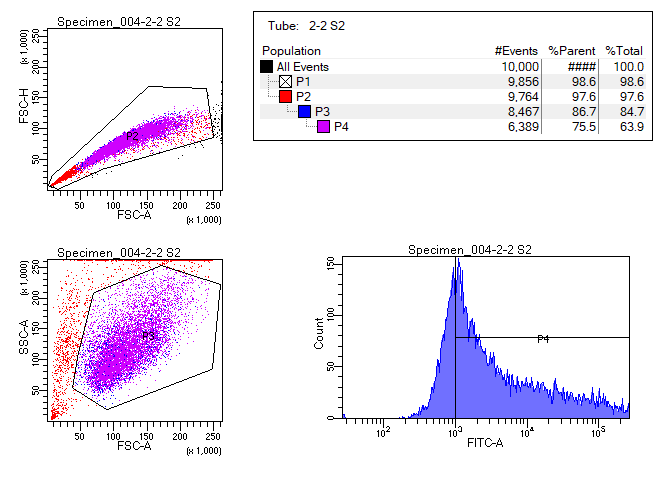


D)


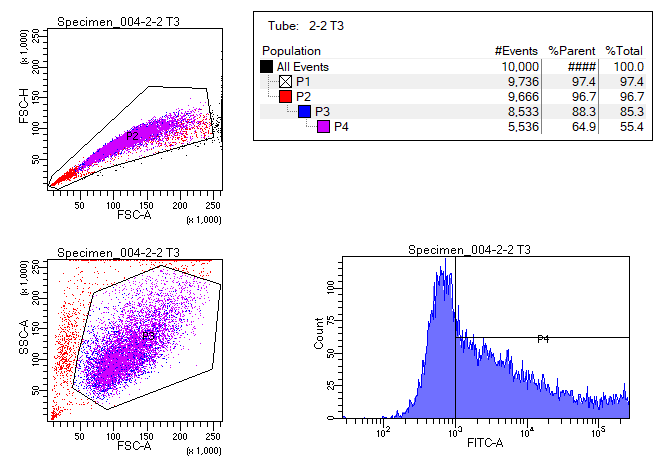


Supplementary Fig. 2.

A)


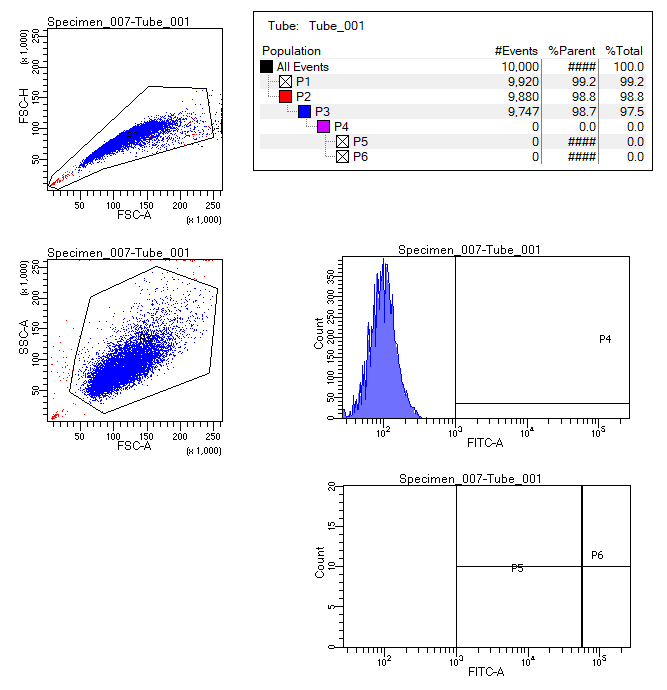


B)


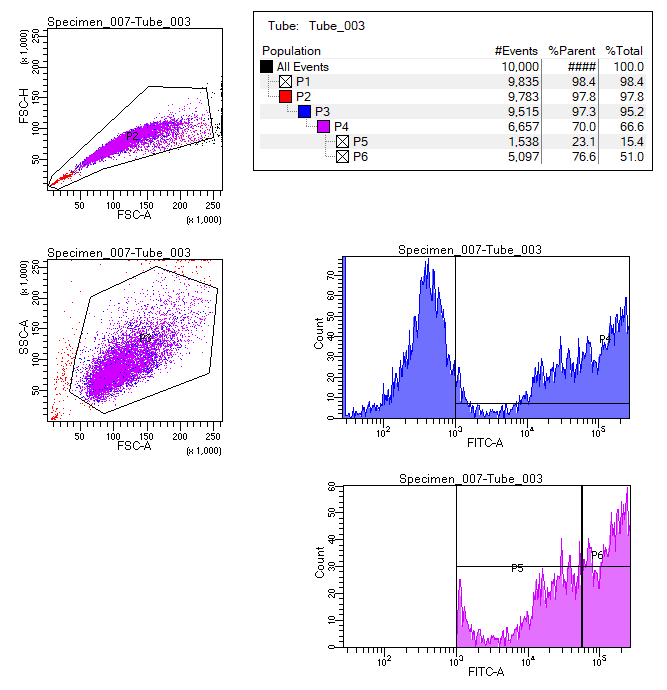


C)


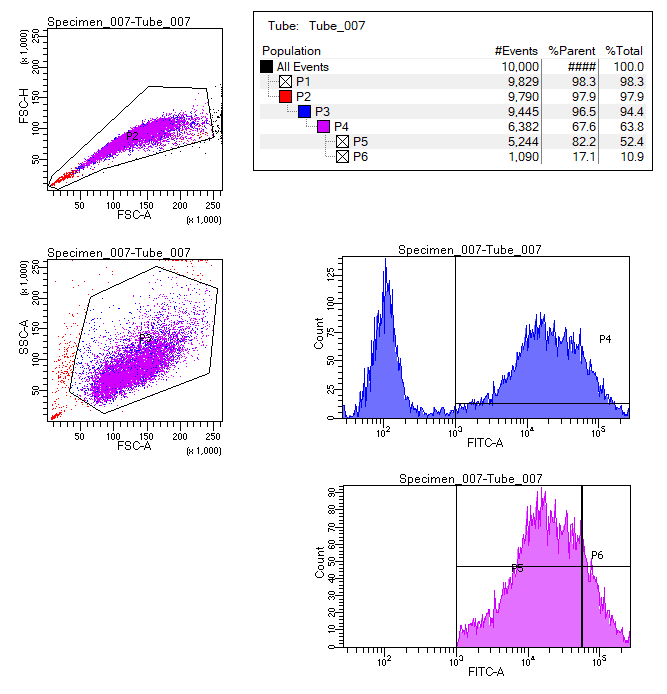


D)


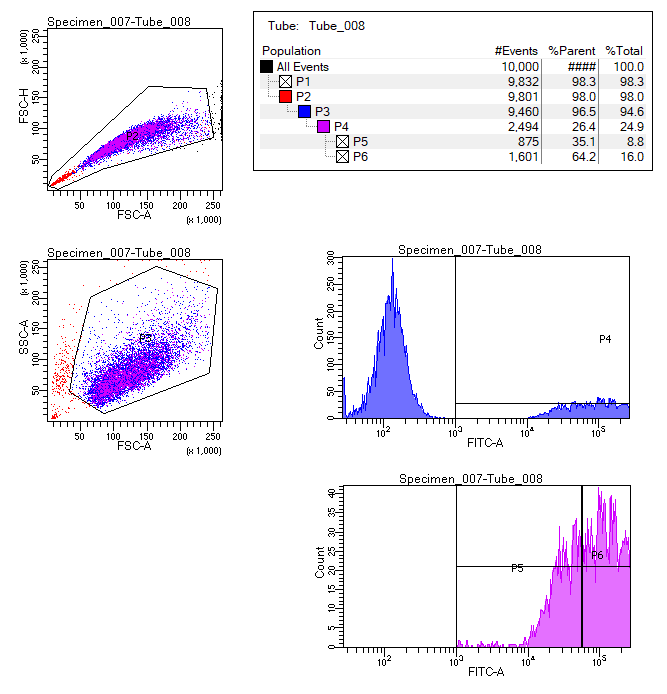


Supplementary Fig. 3.


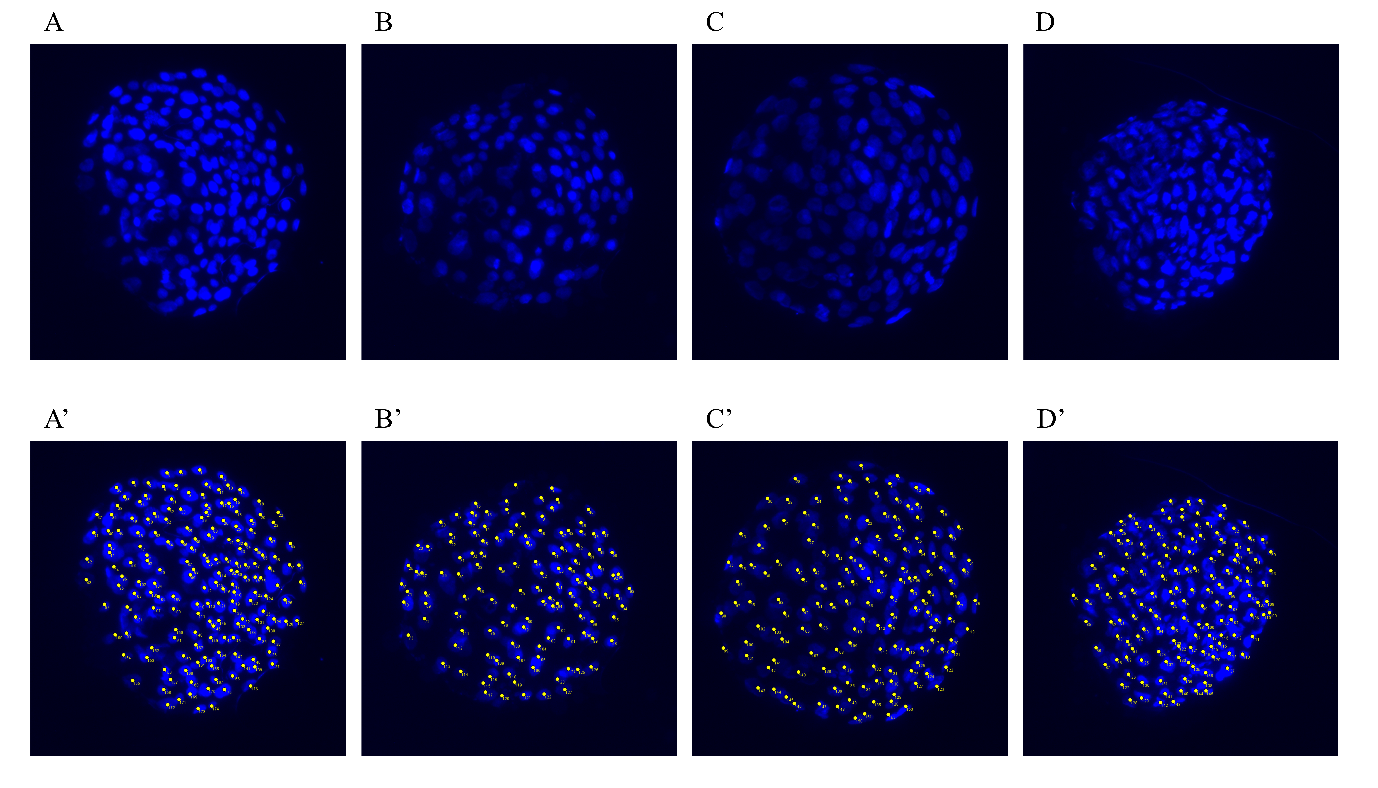

Supplement: Supplementary file 1 — Additional file 1. [file 12917_2022_3252_MOESM1_ESM.docx]
